# Subtle Cardiovascular Dysfunction in the Unilateral 6-Hydroxydopamine-Lesioned Rat

**DOI:** 10.4061/2010/427810

**Published:** 2010-03-21

**Authors:** K. Slack, R. Billing, S. Matthews, H. N. Allbutt, R. Einstein, J. M. Henderson

**Affiliations:** Department of Pharmacology, Bosch Institute and School of Medical Sciences, University of Sydney, NSW 2006, Australia

## Abstract

The present study evaluated whether the unilateral 6-hydroxydopamine (6-OHDA) model of Parkinson's disease produces autonomic deficits. Autonomic parameters were assessed by implanting a small radiofrequency telemetry device which measured heart rate variability (HRV), diurnal rhythms of heart rate (HR), core body temperature (cBT) and locomotor activity (LA). Rats then received 6-OHDA lesion or sham surgery. 6-OHDA lesioned rats exhibited head and body axis biases, defective sensorimotor function (“disengage” test), and prominent apomorphine rotation (all *P* < .05 versus controls). Diurnal rhythm of HR was lower for 6-OHDA lesioned rats (*n* = 8) versus controls (*n* = 6; *P* < .05). Whilst HR decreased similarly in both groups during the day, there was a greater decrease in HR for the 6-OHDA lesioned rats at night (by 38 b.p.m. relative to 17 b.p.m. for controls). LA and cBT did not differ between surgery groups. This study indicates the unilateral 6-OHDA model of PD shows subtle signs of cardiovascular autonomic dysfunction.

## 1. Introduction

Parkinson's disease (PD) is a progressive neurological disorder, characterized by the symptoms of resting tremor, rigidity, postural instability, gait disturbances, and slowness of movement [1, 2]. Overt clinical symptoms of the disease are not apparent until there is a loss of 70% of nigral neurons and 80% of striatal dopamine [3, 4]. The dopamine precursor, levodopa continues to be the main treatment of PD, and it is highly effective for treating the motor symptoms. However, current treatments are ineffective in treating the other disruptive symptoms of the disease [5, 6]. Recent studies have reported that up to 80% of PD patients experience autonomic dysfunction to varying degrees [7–9] of which cardiovascular symptoms attract the most clinical interest as these are the most likely to lead to serious complications, even death [7]. Other autonomic symptoms of PD include gastrointestinal and thermoregulatory/sudomotor symptoms, urinary and sexual dysfunction, as well as sleep and respiratory disorders [7–9]. There are also alterations in the circadian rhythms of blood pressure with greater diurnal variability and the presence of postprandial hypotension [10]. Furthermore, there is a growing body of experimental and clinical evidence suggesting that the basal ganglia plays not only a role in the control of motor function, but also autonomic activity [9].

Animal models have been widely used to give insights into the pathophysiology of PD (for reviews, see [11, 12]). In the rat, the dopaminergic substantia nigra (SN) may be destroyed by lesioning the nigrostriatal tract via the medial forebrain bundle (MFB) using an analogue of dopamine, 6-hydroxydopamine (6-OHDA). This is the most common model and has been found to effectively mimic the degree of dopaminergic cell loss and some motor symptoms of PD [11–13]. 

Limited recent studies have shown that animal models of PD do exhibit some autonomic dysfunction, such as alterations in diurnal rhythm (decreased thermoregulatory function), sleep disorders, and dysfunctions in blood pressure regulation [14–16]. However, these studies used bilateral 6-OHDA animal models of PD, and lesioned a different area to the MFB [14–16]. The bilateral model is less commonly employed by researchers as the animals generally exhibit severe aphagia, adipsia, loss of body weight, and greatly increased mortality during the day after surgery [11], serious health issues which do not occur in the unilateral animal model [17]. A bilateral striatal lesion model, however has been created which exhibits >80% striatal dopamine depletion and minimal mortality [18]. The unilateral 6-OHDA model is used in the present study as it is the most widely utilised model for the experimental study of PD in rats [19]. Whilst one study found an increase in Q-T interval via short-term ECG [19], to date, no studies have performed chronic telemetric analysis assessing heart rate variability or diurnal rhythm in this model. For these reasons, the present study was designed to assess whether rats with a large unilateral 6-OHDA lesion of the MFB exhibit evidence of autonomic deficits. The presence of autonomic deficits was assessed by implantation of a small radiofrequency telemetry device which measured heart rate (HR), core body temperature (cBT), and locomotor activity (LA). Telemetry has previously been shown to be an effective measure of autonomic nervous system activity in the rat [14, 15, 20].

## 2. Methods

### 2.1. Animals

Forty Female Sprague-Dawley rats, weighing approximately 200–260 g and aged 10–12 weeks old on arrival, were housed in 20 pairs (cages: 30 × 40 × 16 cm), with standard rat chow and water available ad libitum. Temperature was controlled at 21.5 ± 1°C, with a light-dark cycle (12:12 hrs, light from 07:00 to 19:00, dark from 19:00 to 07:00). The experiments were conducted with ethical approval from the Animal Care and Ethics Committee of the University of Sydney, Australia, and conducted in accordance with Australian National Health and Medical Research Council's Guidelines on the Use and Care of Animals in Research (1997).

### 2.2. Experimental Timeline

**Figure 1:**
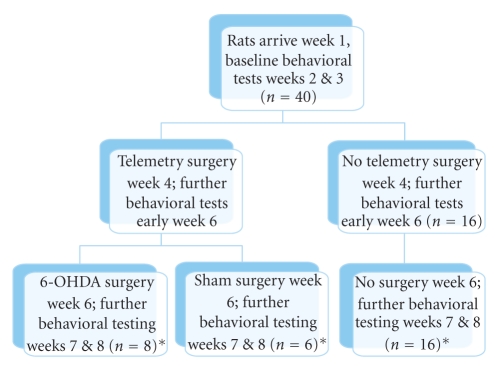
Flow chart illustrating study design. Note that whilst 40 rats were employed, 10 were excluded due to either surgical complications or mistargeting of the 6-OHDA lesion. *final number employed in data analysis.

Rats were acclimatised for one week with gentle handling before beginning behavioral testing for a period of 2-3 weeks. The rats weighed approximately 285 g at the time of telemetry surgery and subsequent unilateral 6-OHDA lesions. The heavier of the two cagemates underwent telemetry surgery (week 4). Rats were given one week to recover from surgery (week 5), after which postoperative measurements of behavioral parameters and telemetry data were recorded for a week (week 6). 6-OHDA lesion surgery was then performed, and a further two weeks of behavioral testing was carried out (week 7 & 8) during which telemetry data was also recorded (Figure 1). 

### 2.3. Techniques

#### 2.3.1. Behavioral Testing

Tests were carried out once per week as (1) baseline measurements for 1–3 weeks before telemetry surgery; (2) measurements one week after telemetry surgery; then, (3) for two weeks following MFB surgery. These tests were carried out on both control and lesioned animals with each animal being tested in a clean test cage.

**(1) Observation of Head Position**

Briefly, the rat was allowed to habituate to the test cage, and head position (>10° deviation left or right from midline, or neutral) was noted every second for 60 s in triplicate following the tick of a watch [13, 21]. Net head position bias was calculated as the total number of seconds head position was held ipsilaterally minus the total number of seconds head position was held contralaterally over a total of 180 s [13, 21].

**(2) Curling**

This test is a modification of the elevated body swing test, as described previously [21, 22]. Briefly, rats were gently suspended for 5 s by the base of the tail over the empty test cage floor with the paws 2 cm above the floor. Curling direction bias was noted as a deviation of ≥10° from the vertical axis. An ipsilateral turn was recorded as +1, a contralateral turn as −1, and no turn as 0. Severity was expressed on a scale of 0 to 3 (i.e., 0 = no curling, 1 = mild (10–60 degrees), 2 = moderate (>60–120 degrees) and 3 = severe (>120 degrees) curling). Testing was performed in triplicate on each test week.

**(3) Sensorimotor and Disengage**

This test was carried out as previously described to measure sensorimotor deficit as a result of nigrostriatal dopamine depletion induced by the 6-OHDA lesion [21]. The left and right vibrissae were touched by a probe, and the sensorimotor response (conducted in the absence of chocolate) latency was measured using a stopwatch and involved either moving or touching the probe with mouth, snout or paw [21]. This was then repeated for the “disengage” test whilst the rat was eating a 2.5 g piece of chocolate [13].

**(4) Apomorphine Rotation**

Rats were injected with apomorphine to test rotational asymmetry [21]. Prior to sacrifice, rats were injected subcutaneously with 0.2 mg/kg apomorphine hydrochloride (Sigma, Steinheim, Germany) dissolved in 1% ascorbate saline solution. 10 minutes after injecting, rats were placed into a white, hemispheric, plastic rotation bowl (42 cm wide at top and 22 cm deep) and recorded for 30 minutes using a DVD camera. The recording was manually scored and net contralateral turning bias calculated (number of contralateral minus number of ipsilateral turns) [11].

#### 2.3.2. Telemetry

**(1) Telemetry System**

HR, LA, and cBT were recorded by means of a telemetry system. This consisted of an implantable radiotransmitter (model TA11-CTA-F40, DataSciences International, St. Paul, MN, USA), a radio receiver (model RPC-1, DataSciences) to receive telemetered signals, a data exchange matrix to multiplex multiple cage signals to the computer, and a computer-based data acquisition system (Dataquest Silver version 3.0, DataSciences). The receiver detected the radio waves and rodent activity as counts and also the calibrated cBT, data which were registered in the computer system.

**(2) Telemetry Surgery**

Transmitter implantation was carried out under aseptic conditions with some modification of the technique described by Kramer and colleagues [23]. 18 rats were anaesthetised with a mixture of 75 mg/kg ketamine and 10 mg/kg xylazine (i.p.) and placed on a sterile drape covering a heating mat (physiological temperature), to prevent hypothermia. Briefly, a midline incision of 2-3 cm was made, with two more incisions (1 cm each) made at the upper right chest and lower left leg to expose the muscle tissue. The transmitter was inserted into the peritoneum and the ECG leads extended subcutaneously to the right upper rib area and the lower left leg and sutured to muscle tissue there. The skin (abdomen, leg, and chest) was sutured closed with absorbable suture and cleaned. Prophylactic broadspectrum antibiotics (5 mg/kg enrofloxacin, s.c.) and pain relief (0.05 mg/kg buprenorphine, s.c.) were administered, and warmed sterile saline (2. mL, i.p.) was administered to replace fluid lost during surgery. Rats were intensively monitored during the next 24 hours postoperatively for any adverse effects.

#### 2.3.3. Unilateral 6-OHDA Lesions

Eighteen rats underwent stereotactic surgery, as described previously [13, 21]. Rats (*n* = 7) were initially anaesthetised using a mixture of ketamine/xylazine (75 mg/kg ketamine hydrochloride Ketavir, Delvery, NSW, Australia plus 10 mg/kg xylazine hydrochloride, Ilium Xylazil-20, Troy Laboratories, NSW, Australia, given i.p.) but due to better control of depth of anaesthesia the remainder were anesthetised using isoflurane (4% induction, 2% maintenance dose, *n* = 11) in oxygen (1.5 L/min). Rats were secured in a Stoelting stereotactic frame (Lab Standard model 51 600, Stoelting Co., Wood Dale, IL, USA), using 45° nonpuncture earbars with the nose bar positioned 2.3 mm below the interaural line. 4 *μ*g/*μ*L of 6-OHDA (H 8523, Sigma-RBI, St. Louis, MO, USA) in 0.1% ascorbate saline solution, was delivered at a rate of 1 *μ*L/min for four minutes (via a 25-26 gauge Hamilton microsyringe mounted vertically onto the stereotactic frame) unilaterally into the medial forebrain bundle (MFB) in 12 rats (co-ordinates: AP −4.4 mm and L ±1.1 mm from bregma, V −8.0 mm from dura) [24]. Sham operated controls (*n* = 6), received only vehicle solution. Buprenorphine (Temgesic, Reckitt-Benckiser, Hull, UK) was given prophylactically for analgaesia (0.05 mg/kg s.c.).

#### 2.3.4. Collection of Tissue

**(1) Histology**

Two weeks after 6-OHDA surgery the rats were culled by terminal anaesthesia with halothane, then perfused with 0.9% saline followed by 4% paraformaldehyde in phosphate buffered saline (PBS). Brains were rapidly removed from the skull and placed into paraformaldehyde (4% buffered solution) overnight. They were transferred to 30% sucrose solution for 2 days for cryoprotection. Brains were sliced on a Leitz 1320 (Wetzler, Germany) freezing sledge microtome at 48 *μ*m intervals, generating 5 series. One series was mounted onto gelatinised slides and stained for cresyl violet, which allowed visualisation of the needle tract. A parallel series was used for confirming 6-OHDA lesions by immunohistochemical staining of the tissue with tyrosine hydroxylase, as described previously [13]. Multiple serial sections were viewed under a binocular microscope at 20× using a 10× objective. This enabled loss of both dopaminergic nigral and striatal terminals to be visualised. Size of 6-OHDA lesions was quantified by viewing multiple serial TH-stained sections (spaced 240 *μ*m apart) throughout the entire extent of the nigra under a microscope using a 10 × 10 grid eyepiece (of 2.49 mm^2^ area). The total neuronal number was estimated bilaterally. By comparing TH-stained cell numbers between the lesioned and intact side of the brain, the % cell loss (lesion size) was calculated [13].

### 2.4. Data Analysis

#### 2.4.1. Telemetry Data Acquisition and Analysis

Telemetry data was collected 24 hours a day, for 10 s every 5 minutes, using the Dataquest acquisition program. For diurnal rhythm data of HR, LA, and cBT, data was processed in Excel and an average was calculated for day/night periods (light/dark, 12:12 h). Data was taken from 3 days before the 6-OHDA lesion/sham MFB surgery and for a further 13 days afterwards. Amplitude (night minus day) was calculated from this data, and for this postlesion data was normalised as a percentage of mean prelesion data. 

HR data continuously sampled for the 24 hours period prelesion and a 24 hours period in the second week postlesion was compared. Offline ECG analysis was performed using Chart version 5 (AD Instruments, Australia), with digital filtering of 20–40 Hz.

#### 2.4.2. HRV Analysis

24 hours periods of data pre- and postlesion data were divided into 12 hours periods, to compare day-night rhythms. Before analysis, data was collated in Chart and artefact removed to reduce potential sources of error (e.g., extra detection of RR intervals [25]).

#### 2.4.3. Time and Frequency Domain Analysis

Time domain parameters quantified were (1) the mean N-N interval duration (i.e., the average time distance between two consecutive heart beats (NN, ms)), (2) average heart rate (b.p.m.), (3) the standard deviation of NN (SDNN, ms), and (4) the root-mean-square of successive R-R interval differences (RMSSD, ms). HRV parameters are derived from direct measurements of normal-to-normal (N-N) R-R. SDNN is the simplest estimate of overall HRV. It is a global parameter of variability, which reflects all the cyclic components responsible for variability in the period of recording (including short-term low frequency and high frequency variations) [26, 27]. RMSSD is another common measure [27]. 

Frequency domain methods provide analysis of power spectral density, which previously has been successfully used to analyse autonomic nervous system function in rats [20]. HRV spectral bands were defined as very low frequency (VLF, 0–0.2 Hz, ms²), low frequency (0.2–0.9 Hz, ms²), and high frequency (0.9–2 Hz, ms²). Total power and the LF-HF ratio were also calculated. The latter is considered to be a convenient index of parasympathetic and sympathetic interactions in the rat, whilst total spectral power ranges from 0.0 to 2 Hz and reflects the global spectral density [28].

#### 2.4.4. Chronobiology Analysis of Heart Rate

Data from day and night recordings were combined to give 24-hour periods for frequency analysis. An hourly average of a 10 second recording of HR was obtained for chronobiological analysis. Fourier spectrum analysis using Microsoft Excel Fourier Analysis was performed to establish the dominant frequency and its' periodicity.

The single cosinor method was used to fit a cosine function to the values which determined mesor (mean value), amplitude (nadir difference), and acrophase (hour of detection of the peak) for the recordings as previously detailed [14].

#### 2.4.5. Statistical Analysis

Data was statistically analysed using StatView version 5 (SAS, USA). *P* < .05 was taken as significant. Results are expressed as mean ± SEM.

Head position, curling, and sensorimotor and disengage tests were each analysed using two-way repeated measures analysis of variance (ANOVA) and post hoc Scheffe's test for group differences [13]. ANOVA was used to detect differences in the magnitude of dopaminergic cell loss between groups. Apomorphine rotation test was analysed via student's *t*-test (6-OHDA lesioned vs pooled control groups) [13].

Normalised amplitude and data for time- and frequency-domain measures was statistically analysed in StatView using repeated measures of variance (ANOVA) and Scheffe's post hoc test. Postlesion data for diurnal rhythm was normalised by taking a mean of the prelesion data, and calculating the postlesion data as a percentage of this. ANOVA was performed for data pre- and postlesion, for day and night data and for a 24-hour period. Mesor HR values were compared statistically for all animals using 2-way repeated analysis of variance by surgery type (6-OHDA *n* = 6 and Sham lesioned *n* = 4) and pre/postlesion measures for both day and night recordings. However, there were too few significantly periodic results for statistical comparison of either of the wave components, amplitude, and acrophase.

## 3. Results

### 3.1. Animal Numbers

From the forty animals that were used, ten animals had to be excluded due to surgical complications (*n* = 6; of these 4 developed infections of the abdominal wound site associated with repetitive picking of stitches and 2 developed respiratory infections; all of which were euthanased) or inadequate 6-OHDA lesioning (*n* = 4 with <70% nigral cell loss; see below). This left 8 × 6-OHDA lesioned, 6 sham lesioned (all rats in these groups received telemetry implants) and 16 normal (unoperated cagemate) rats.

### 3.2. Quantification of Lesions

**Figure 2:**
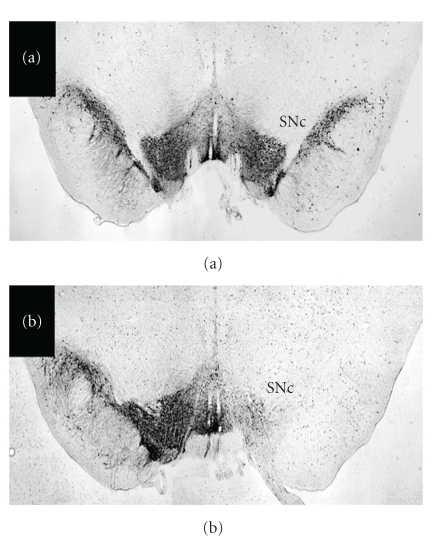
Photomicrographs of tyrosine hydroxylase immuno-stained nigra of a control (a) and 6-OHDA lesioned (b) rat. Images are representative of the effect of a saline/sham microinjection (a) and 6-OHDA lesion (b) of the MFB on the SNc. The nigral DA neurons are highly TH-immunoreactive; note the lack of staining on the right side of (b), indicative of a large lesion of the dopaminergic SNc. In contrast, there is preservation of the nigra on the targeted (right) side in (a). MFB, medial forebrain bundle; SNc, substantia nigra pars compacta.

Analysis of TH-immunoreactivity in the SN (see Figure 2) indicated that eight of 12 lesioned with 6-OHDA had complete lesions. Sham-lesioned rats (*n* = 6) had no significant nigral cell loss and cresyl violet staining confirmed correct MFB targeting.

### 3.3. Behavioral Testing

All animals (implanted and cagemates) were tested prior to telemetry surgery, and no differences were found between groups in all behavioral tests. No differences were found following telemetry surgery, showing that implantation of the device had no influence on the behavioral tests. After MFB surgery, there remained no difference between sham surgery (saline-injected *n* = 6) and untreated normal cagemates (*n* = 16), therefore data for these two groups was pooled (*n* = 22 controls) for analysis of behavioral testing data. There was a marked difference between the 6-OHDA lesion animals versus pooled control group in all four behavioral tests.

#### 3.3.1. Behavioral Confirmation of Parkinsonism after 6-OHDA Lesions

**(1) Postural Measures**

**Figure 3:**
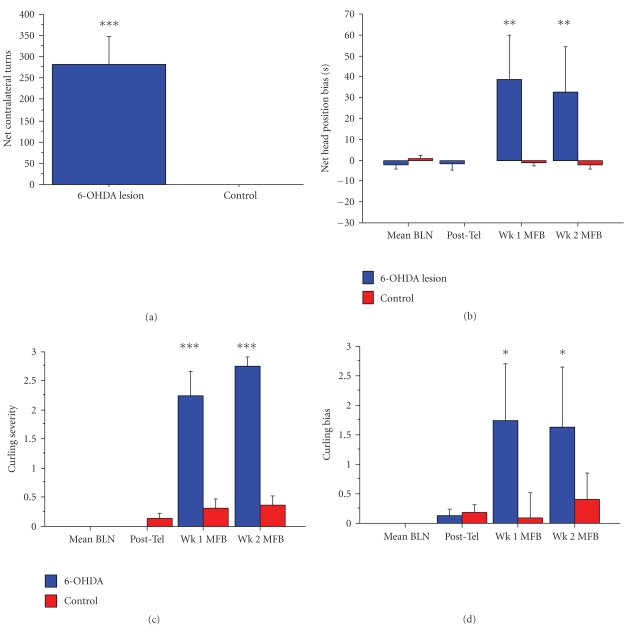
Behavioral parameters. (a) Apomorphine rotation test. Net contralateral turning bias recorded as number of turns made in a 30 minutes period. Apomorphine induced an overt contralateral turning bias in the 6-OHDA lesioned group, but had no effect on the pooled control group, which did not exhibit rotation (unpaired *t*-test: *t* = 6.349, ***P < .0001). (b) Head position bias. There was no bias prior to MFB surgery. 6-OHDA lesions induced an ipsilateral bias in head position in contrast to pooled controls which showed no bias (head bias ANOVA: *F* = 6.983, *P* = .0003, post hoc group difference *P* = .0006). **P < .001. (c) Curling severity and (d) direction of bias. There was no bias at the baseline. 6-OHDA lesions induced an ipsilateral bias in curling in contrast to pooled controls which showed negligible bias (curling severity ANOVA: *F* = 79.872, *P* < .0001; post hoc group difference *P* < .0001; curling bias ANOVA: *F* = 3.400, *P* = .0215; post hoc group difference *P* = .0398). Vertical bars indicate mean ± SE of each group. BLN, baseline; MFB, medial forebrain bundle. Post Tel, posttelemetry device implantation. Wk 1, Wk 2, weeks 1 and 2 after MFB surgery, respectively. ***P < .0001; *P < .05.

After MFB surgery, the pooled control group continued to exhibit no head position or body axis bias, whereas there was a marked ipsilateral bias in these parameters in the 6-OHDA lesioned group (see Figure 3). For the apomorphine rotation test, a marked contralateral turning bias was seen in the 6-OHDA lesion group whereas no rotational asymmetry was noted in the pooled control group (Figure 3).

**(2) Sensorimotor Tests**

Whilst 6-OHDA lesion rats did not exhibit a significant increase in sensorimotor neglect in the simple task on either side after MFB surgery (data therefore not shown) there was prominent contralateral slowing in response time to tactile stimulation in the disengage test relative to pooled controls (ANOVA: *F* = 16.594, *P* < .0001, post hoc: *P* < .0001).

### 3.4. Telemetry Data

Telemetric data was derived from all 14 rats with successfully targeted MFB surgery. Data from a subset of ten operated rats (six, 6-OHDA lesioned and four sham lesioned) was used for further HRV and circadian analysis as a result of technical problems with acquisition in four rats.

#### 3.4.1. Diurnal Rhythm

**Figure 4:**
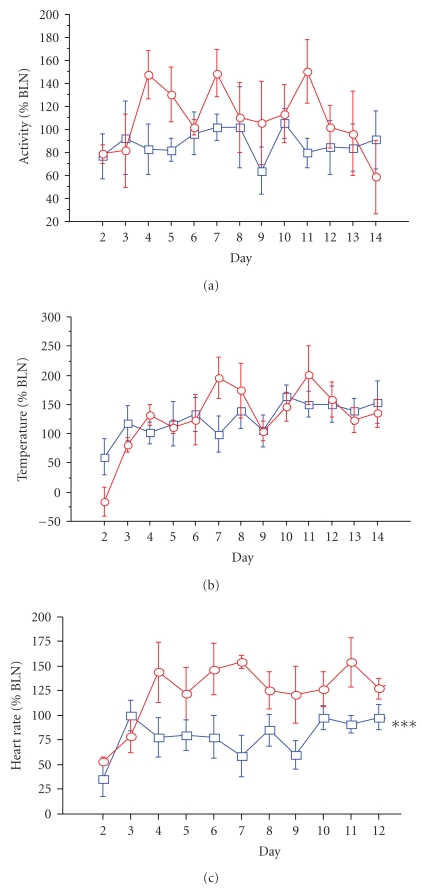
Circadian rhythm data. Normalised data for amplitude values were calculated as post-MFB surgery values as a percentage of mean prelesion (BLN, baseline) data for the circadian parameters of: LA (a), cBT (b), and HR (c), for sham-operated controls and 6-OHDA lesioned rats. Data was taken from the day after MFB surgery (day 2) until 12–14 days after surgery. Data was analysed from day 4, when recovery of HR and body temperature reached levels comparable with the baseline in the lesioned rats. There was no significant change in LA (a) or cBT (b) between groups. However, there was a lower diurnal rhythm of HR for 6-OHDA lesion rats than sham-operated controls (ANOVA: *F* = 12.280, *P* = .0057; post hoc group difference ***P < .0001). Circles represent controls; boxes represent 6-OHDA lesioned rats. BT, body temperature, HR, heart rate, LA, locomotor activity.

After telemetric implantation, prelesion data was taken when recovery of HR and body temperature reached levels comparable with the baseline in the lesioned rats. Post-MFB surgery data was therefore taken from day 4 after surgery until approximately day 12–14 (Figure 4). There was no significant change in LA (Figure 4(a)) or cBT (Figure 4(b)). However, there was a lower diurnal rhythm of HR for 6-OHDA lesion rats than sham-operated controls (Figure 4(c)). For the night and day diurnal rhythm data, there was an overall decrease in HR after MFB surgery. While there was a similar decrease in HR for both groups during the day (by 19 b.p.m. for 6-OHDA lesion rats, relative to 21 b.p.m. for sham-operated controls), there was much greater decrease in HR (approximately two-fold) from baseline for the 6-OHDA lesioned rats in the night compared to sham-operated controls (by 38 b.p.m. versus 17 b.p.m., resp., *P* < .05). 

#### 3.4.2. Heart Rate Variability

Data was compared for night and day recordings (12:12 h) for pre- and postlesion, and for 24 hours recordings pre- and postlesion (data taken between 11–14 days after telemetry surgery and MFB surgery). Night versus day recordings were not significantly different between surgery groups. However, analysis of the 24 hours recordings revealed differences in time domain and spectral parameters.

**(1) Time Domain Parameters**

There was a similar decrease in average HR after MFB surgery in both groups. 6-OHDA lesion rats had a decrease in mean HR of 20 b.p.m. from prelesion to postlesion which was similar to controls which had a decrease of 30 b.p.m. (average HR in 24 hours recording, pre- versus postlesion: ANOVA: *F* = 32.522, *P* = .0005; post hoc: *P* = .7183). There was a corresponding increase in mean NN interval (pre- versus postlesion: ANOVA: *F* = 29.044, *P* = .0007; post hoc: *P* = .6565) and SDNN values for both surgery groups (pre- and postlesion: ANOVA: *F* = 8.852, *P* = .0177; post hoc: *P* = .9005; data not shown), indicating that this was a time effect. There was no significant change in RMSSD values in both surgery groups after MFB surgery.

**(2) Spectral Analysis**

Fast fourier transform spectral analysis was performed on ECG recordings from sham control and 6-OHDA lesioned rats. There was no significant difference in pre- and postlesion data for the total power, VLF, LF or HF components of the spectrum. There was a similar increase in mean LF : HF ratio in both groups after MFB surgery (LF : HF ratio for 24 hours recordings, pre- and postlesion : ANOVA: *F* = 9.574, *P* = .0148; post hoc: *P* = .9608; data not shown).

**(3) Chronobiological Analysis of Heart Rate**

**Figure 5:**
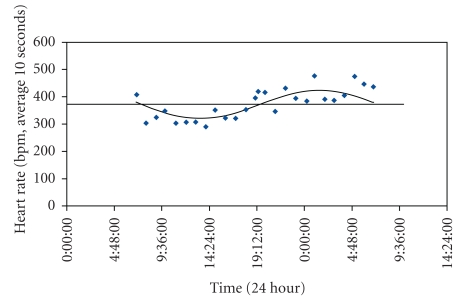
Example of fitted circadian rhythm in a representative rat for 24-hour heart rate post-Sham surgery. Cosinor analysis revealed significant periodicity (*P* < .05); with mesor = 372.2 (horizontal line above), amplitude = 51.6, acrophase = 1 : 28 hours. bpm = beats/min.

Fast Fourier spectrum analysis revealed a dominant frequency corresponding to a approximately a 24-hour wavelength in >90% of the 24-hour datasets where periodicity was present. Significant periodicity occurred in 7/12 (58%) and 5/8 (63%) of the 24 hours periods sampled in the 6-OHDA lesioned and sham-operated groups, respectively. An example of a fitted curve for a representative rat which exhibited such periodicity is given in Figure 5. When examining 24-hour recordings from the two surgical groups, a significant drop in mesor for HR was evident in sham-operated rats (ANOVA: *F* = 12.75, *P* < .05), unlike 6-OHDA lesioned rats, when compared to the prelesion state (Table 1).

## 4. Discussion

### 4.1. General Observations

There was a greater decrease in HR diurnal rhythm for unilateral 6-OHDA lesioned rats (relative to the controls), consistent with that reported in the bilateral 6-OHDA animal model of PD [14]. This reflected a lower nocturnal HR in hemiparkinsonian rats. There was also a generalized effect of surgery on HR in both sham control and lesioned groups following MFB surgery.

 The behavioral data was consistent with previous studies of parkinsonism in the unilateral 6-OHDA animal model of PD. Large unilateral depletions of nigrostriatal DA in rats leads to ipsilateral biases in the curling and head position tests [13, 21, 29, 30], contralateral deficits in the disengage task [13, 31], and marked contralateral apomorphine-induced rotation [32], suggesting that the rats were adequately lesioned. The degree of lesioning was also consistent with what we have previously observed in the same gender and strain of rats, providing internal consistency between studies [13]. Furthermore, there was an almost complete loss of DA nigral neurons in 6-OHDA lesioned rats on the lesioned side. This ensured that the animal model created was sufficiently parkinsonian and therefore appropriate for comparative telemetric analysis.

### 4.2. Choice of Model Relative to Other Studies

The novelty of this project was that the (1) unilateral 6-OHDA rat model of PD had never been used in a telemetric study of autonomic dysfunction and (2) previous studies of autonomic dysfunction have not targeted the MFB (i.e., the striatum was lesioned bilaterally or the ventral tegmental area, VTA itself was targeted [14, 15]). Given that the unilateral 6-OHDA lesioning of the MFB is the model most commonly used in PD research, it was of relevance to examine this issue. Since there is no striatal neurodegeneration in PD and the VTA (A10) is not the major site of dopaminergic cell loss in patients [33], targeting the MFB produces an animal model more likely to mimic nigral degeneration and accompanying motor symptoms of PD than models targeting either VTA or the striatum. Striatal DA depletion from nigral degeneration leads to increased inhibition of the motor thalamus, by causing glutamatergic subthalamic hyperactivity and increased GABAergic transmission via basal ganglia output nuclei (SN pars reticulata/medial globus pallidus) [34]. The decreased thalamocortical activation produces the slowness of movement (bradykinesia) and rigidity characteristic of PD [34]. A major ethical advantage of the unilateral rat model used in this study is that the rats suffer less morbidity (can eat and drink independently within hours of surgery) and mortality than the bilateral model (i.e., can eat and drink independently within hours of surgery and have 97% versus 50% survival rate, resp.) [35, 36].

### 4.3. Telemetry

#### 4.3.1. Heart Rate

The main finding from telemetry was a lower amplitude of diurnal rhythm for HR in 6-OHDA lesion rats compared to sham controls (i.e., the difference between the day: night or light: dark cycle). This was due to the decreased HR of 6-OHDA lesion rats during their night/wake cycle. Ben and Bruguerolle [14] also found a decrease in the amplitude for HR diurnal rhythm in four rats with a double bilateral 6-OHDA lesion of the striatum relative to three sham-operated controls during telemetry for three weeks postoperatively [14]. However, there was >30% loss of body weight in the parkinsonian rats suggesting poorer health which could adversely impact on autonomic measures [14]. The dopaminergic innervation of the striatum was affected in the present study by the lesion of the nigrostriatal tract. Both studies suggest a relationship between the loss of DA in the striatum and the control of HR. Previous studies have found evidence to support a direct association between the nigrostriatal system and cardiovascular function [37–39]. Electrical [40] or chemical [41] stimulation of the SN pars compacta in rats enhanced DA release in the striatum and elicited proportional hypertension and tachycardia. Furthermore, other studies injected various tachykinin agonists bilaterally into the SN pars compacta and found that in awake, unrestrained rats tachycardia occurred [42], whereas in spontaneously hypertensive rats tachycardia and mean arterial pressure increased [43]. In addition, high frequency deep brain stimulation of the nigra during neurosurgery in PD patients was associated with increased heart rate and blood pressure [44]. These combined data support a role for nigral regulation of both cardiovascular parameters. It could therefore be inferred that loss of DA in this area could lead to hypotension and decreased HR, that is, a decrease of sympathetic innervation in the heart, which are both cardiovascular features of PD [45]. The decrease in the HR for the wake cycle of the 6-OHDA lesion rats may be due to fluctuations in DA metabolism or DA accumulation during the day/sleep cycle, which is a suggested reason for diurnal fluctuations in parkinsonian symptoms [46]. 

Apart from the larger decrease (approximately two-fold greater) in HR for 6-OHDA lesion rats, after MFB surgery HR was somewhat lower for both surgical groups, for both night and day. This indicates that there not only was there a substantial effect of DA cell loss, but there was also a general effect of the surgery itself on HR. Previous research has shown that surgery can blunt the amplitude of diurnal rhythms with the effects on HR and cBT lasting for 12–15 days [47]. This could explain the subtle drop in BT during the day in both groups after MFB surgery. One study [48] monitored the effect of anaesthesia (ketamine or ether) via telemetry and found an effect on diurnal rhythmicity for 7–10 days [48]. Since our surgical groups were matched for anaesthesia type, this factor is unlikely to account for the greater nocturnal decrease in HR in parkinsonian rats but may contribute to the generalized postoperative decrease in HR found in both groups.

#### 4.3.2. Core Body Temperature

Whilst thermoregulatory dysfunction has been reported in PD patients [8], a recent paper specifically examining this issue found that patients with idiopathic PD exhibited a nocturnal fall in cBT similar to controls, whereas parkinsonian patients with multiple system atrophy exhibited an abnormal response [49]. In the present study, whilst there was no overall difference between the 6-OHDA versus sham-lesioned rats from day four, in the first couple of days postoperatively a lower cBT was evident in the 6-OHDA lesioned rats (Figure 4(a)). A previous study also noted a transient drop in cBT for a few days after bilateral striatal lesions accompanied by a loss of periodicity of the circadian rhythm for this parameter for a month postoperatively [14].

#### 4.3.3. Locomotor Activity

No significant effect was found in the amplitude of the diurnal rhythm of locomotor activity following MFB lesioning, similar to the bilateral lesion rat model [14]. This contrasts with another study reporting that LA itself was suppressed following a lesion to the ventral tegmental area (VTA) [15]. Differences in surgical targeting may account for this.

#### 4.3.4. Heart Rate Variability

The significantly altered HRV in both groups after MFB surgery, indicates a general effect of surgery on cardiovascular function, at least in the first fortnight after surgery. Analysis of HRV has been found to be an effective indirect measure of variability in ANS activity [50]. HRV describes both instantaneous heart rate and the beat-to-beat heart rate signal (R-R interval) [27]. Lower variability is often an indicator of abnormal and insufficient adaptability of the ANS, implying the presence of a physiological malfunction [51]. PD patients exhibiting cardiovascular complications have decreased HRV [52] which is a sensitive and specific predictor of mortality [53].

#### 4.3.5. Time and Frequency Domain Analysis

In the time domain analysis, there was a notable increase in the HRV parameters of mean NN interval and SDNN (as compared to baseline) reflecting a shift of sympathovagal balance towards a parasympathetic prevalence [54]. For frequency domain analysis, there was a marked increase in the LF/HF ratio, suggesting a predominance of sympathetic rather than vagal activity (LF is mediated by sympathetic activity; HF is parasympathetic activity) [8]. This apparent conflict between time-domain and frequency domain analyses has also been observed in unrestrained rats and it has been suggested that frequency domain measures are more suitable for investigating parasympathetic and sympathetic interactions in the rat [20]. It is also generally agreed that initial cardiovascular symptoms of PD are parasympathetic in nature, and both sympathetic and parasympathetic at later stages of the disease [55]. The unilateral 6-OHDA model used would approximate advanced PD (where there is >90% DA cell loss).

#### 4.3.6. Chronobiology Analysis

Whilst chronobiology analysis was not a major focus of the present study, a subanalysis of telemetry data for heart rate was attempted for comparative purposes [14]. Since approximately 40% of 24-hour samples analysed did not exhibit evidence of periodicity, this limited the analysis. However where present, this approximated a circadian (24 hours) pattern. Whilst a significant effect (drop in mesor for HR) was evident when comparing pre- versus post-MFB surgery conditions in sham-operated rats, this was not evident in 6-OHDA-lesioned rats. The discrepancy between this and the lower amplitude of diurnal rhythm in HR in the 6-OHDA lesioned rats, relative to sham-operated controls is probably accounted for by the different nature of data analysis techniques (mesor versus mean; 24 hours versus day and night data), small numbers of rats and lack of periodicity in approximately 40% of the recordings. The latter probably also explains why we could not detect a significantly lower mesor for HR in 6-OHDA lesioned animals compared to a previous study [14].

Overall this study established that the unilateral 6-OHDA model of PD displayed subtle cardiovascular autonomic dysfunction with reduced nocturnal HR. Where periodicity was present, it followed a 24-hour circadian rhythm for HR. Further studies are required to explore the chronicity of this phenomenon and effects of dopaminergic therapy.

## Figures and Tables

**Table 1:** Average mesor level heart rate values (± SEM) for the two groups. BPM = beats/min.

Average Mesor Level Heart Rate (BPM)			
		Sham	6-OHDA
Prelesion	24 hr	378 ± 10	367 ± 6
Post lesion	24 hr	345 ± 12	364 ± 7
